# Femto-DSAEK for deep posterior stromal opacity: a lamellar alternative to PK in failed DMEK

**DOI:** 10.1016/j.ajoc.2026.102552

**Published:** 2026-03-02

**Authors:** Luca Lucchino, Davide Romano, Sabrina Vaccaro, Antonio Moramarco, Francesco Semeraro, Luigi Fontana, Vito Romano

**Affiliations:** a Eye Unit, Department of Medical and Surgical Specialties, Radiological Sciences and Public Health, University of Brescia, Brescia, Italy; b Department of Sense Organs, Sapienza University of Rome, Rome, Italy; c St. Paul's Eye Unit, Department of Corneal Diseases, Royal Liverpool University Hospital, Liverpool, United Kingdom; d Department of Eye and Vision Sciences, University of Liverpool, Liverpool, United Kingdom; e Ophthalmology Unit, Dipartimento di Scienze Mediche e Chirurgiche, Alma Mater Studiorum University of Bologna, Bologna, Italy

**Keywords:** Femtosecond laser, DSAEK, Corneal stromal scar, Failed DMEK

## Abstract

**Purpose:**

To describe the feasibility of femtosecond laser-assisted stromal dissection combined with Descemet stripping automated endothelial keratoplasty (femto-DSAEK) as an alternative to penetrating keratoplasty (PK) in case of deep posterior stromal scarring following failed Descemet membrane endothelial keratoplasty (DMEK).

**Observations:**

A 58-year-old male with history of failed DMEK in right eye for Fuchs’ endothelial dystrophy was referred at our department for posterior stromal scarring. On examination, the posterior stromal opacity, localized at 501 μm from the epithelium, had a thickness of 80 μm. In view of the above, planning a second DMEK was not expected to yield satisfactory visual outcomes. A femtosecond laser-assisted deep stromal dissection (80 μm depth, 7.50 mm diameter) was performed to selectively remove the posterior stroma, Descemet membrane, and dysfunctional endothelium in a single-step approach. A 7.00 mm pre-cut DSAEK graft, of 82 μm thickness, was subsequently implanted. The procedure resulted in rapid visual recovery and corneal clearing (BCVA improved from 1.00 to 0.10 logMAR), without complications.

**Conclusions and importance:**

Femto-DSAEK offers a streamlined approach for managing failed DMEK with posterior stromal scarring. By enabling precise removal of diseased posterior tissue and immediate endothelial replacement, it may enhance graft adherence, optimize visual rehabilitation, and provide an effective alternative to PK in complex cases.

## 1 Introduction

Posterior lamellar keratoplasty techniques were initially developed to selectively replace diseased endothelium along with a portion of the posterior stroma.^1^ Early procedures such as deep lamellar endothelial keratoplasty (DLEK) involved manual stromal dissection and posterior graft implantation, but were technically demanding and eventually replaced by descemetorhexis-based techniques.^2^ These evolved into current standards such as Descemet Stripping Automated Endothelial Keratoplasty (DSAEK) and Descemet Membrane Endothelial Keratoplasty (DMEK), which avoid stromal dissection by selectively removing Descemet membrane and dysfunctional endothelium.^3^^,^^4^

These modern endothelial techniques are effective in restoring corneal clarity when the underlying stroma remains transparent.^5^ However, in eyes with deep posterior stromal scarring, DSAEK or DMEK alone may not yield satisfactory visual results due to residual haze or poor graft adherence.^6^^,^^7^ In such cases, full-thickness penetrating keratoplasty (PK) is often the only viable option, despite its inherent risks, including higher rates of endothelial rejection and prolonged recovery.^8^

Recent advances in femtosecond laser platforms have revived interest in customized stromal dissection, providing the possibility of selective posterior lamellar excision with high precision.^9^ These technologies enable controlled cuts at user-defined depths and geometries, potentially allowing surgeons to address stromal scarring directly while preserving the anterior corneal architecture.^10^, ^11^, ^12^, ^13^.

There is currently no consensus on the optimal surgical approach in cases of failed DMEK with posterior stromal scarring. This report explores whether a single stage femto-assisted lamellar excision combined with DSAEK could address this surgical challenge.

## 2 Case presentation

**Fig. 1:**
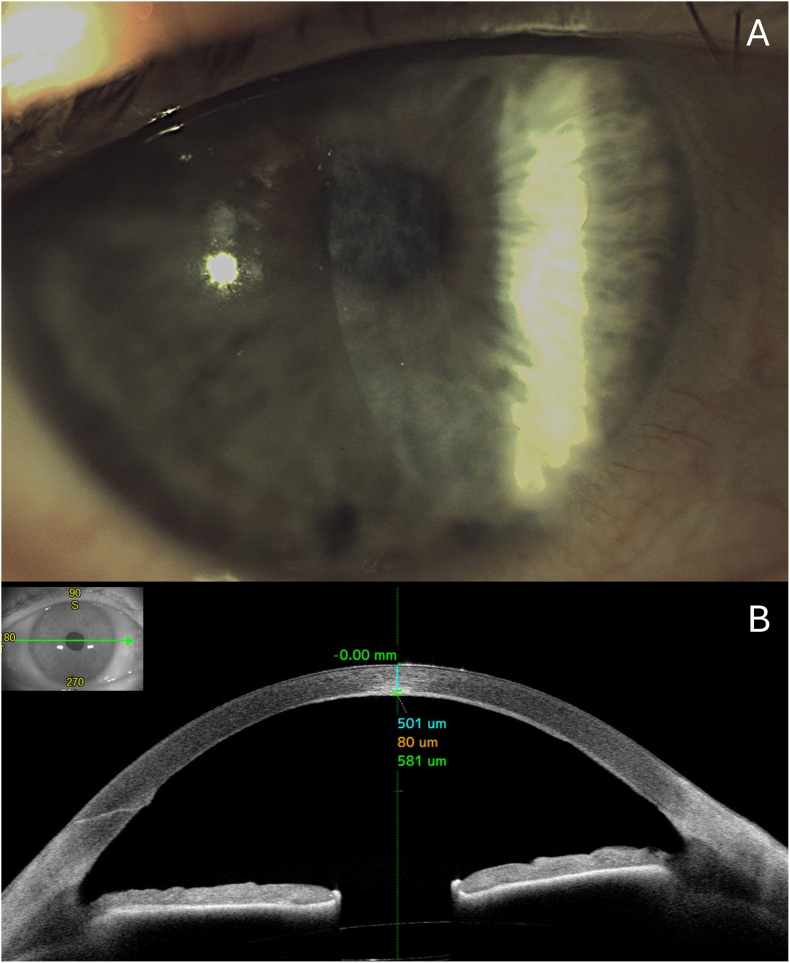
(A) Preoperative Slit-lamp photograph showing posterior stromal haze involving the visual axis. (B) Anterior segment optical coherence tomography (AS-OCT) revealing a hyperreflective stromal scar measuring 80 μm in thickness, located at a depth of 501 μm from the epithelial surface. Total central corneal thickness was 581 μm.

A 58-year-old male with a diagnosis of raised intraocular pressure (IOP), posterior stromal scarring and suboptimal visual recovery following the initial DMEK procedure performed 10 months earlier was referred at our department. Raised IOP was managed with a topical combination of dorzolamide 2% and timol 0.5% twice daily. On examination, best-corrected visual acuity (BCVA) in the affected eye was 1.00 logMAR. Slit-lamp examination was positive for posterior stromal haze, while all other ocular findings were unremarkable (Fig. 1).

To better investigate the depth of stromal scarring, anterior segment optical coherence tomography (AS-OCT; Casia2, Tomey Corporation, Japan) was performed. The total central corneal thickness was 581 μm. The stromal scar appeared as an 80 μm-thick hyperreflective band located at a depth of 501 μm from the epithelium (Fig. 1).

In view of the above, given the presence of residual posterior stromal opacity, repeat DMEK was deemed unfeasible, and PK was initially considered. However, to avoid full-thickness transplantation, the decision was made to perform femtosecond laser-assisted stromal dissection followed by DSAEK (Femto-DSAEK).

The stromal dissection was performed using the Ziemer FEMTO LDV Z8 femtosecond laser (FEMTO LDV Z8, Ziemer, Port, Switzerland), which provides high-precision laser delivery combined with real-time intraoperative OCT imaging to guide dissection depth and centration.

The procedure was carried out in two distinct steps. The first was a horizontal lamellar cut, created at a depth of 501 μm from the anterior corneal surface (above the hyperreflective band in the posterior stroma). This cut defined the stromal plane along which the posterior corneal tissue would later be separated and removed. The depth was carefully chosen based on preoperative pachymetry and confirmed intraoperatively using OCT after docking.

Following this, a vertical dissection was performed, starting from the endothelial side of the cornea and progressing anteriorly until it reached the previously created horizontal cut. This vertical cut effectively isolated a central stromal block, which could then be manually removed. The horizontal dissection was designed to create a precise 7.50 mm diameter cylinder, centered on the visual axis, allowing for a controlled and reproducible stromal resection.

Throughout the procedure, the alignment and depth of the cuts were monitored using the integrated OCT system. Small spatial adjustments (X/Y offset of ±0.35 mm and Z offset of ±0.30 mm) were applied as needed to optimize centration and interface quality.

This approach, combining a posterior-up vertical cut with a posterior stromal lamellar dissection, allowed for a clean and anatomically guided removal of the posterior corneal stroma. The result was a well-defined interface suitable for DSAEK, offering both safety and reproducibility, particularly in complex cases.

A 7.00 mm precut DSAEK graft (82 μm thickness) was then performed as previously described.^14^ A 0.50 mm undersize in DSAEK is used to avoid potential overlap between the host cornea and the donor graft, which could be a risk factor for graft detachment. An intraoperative video showing DSAEK graft insertion and positioning after femtosecond-assisted posterior stromal cut is provided as Supplementary Video 1.

**Fig. 2:**
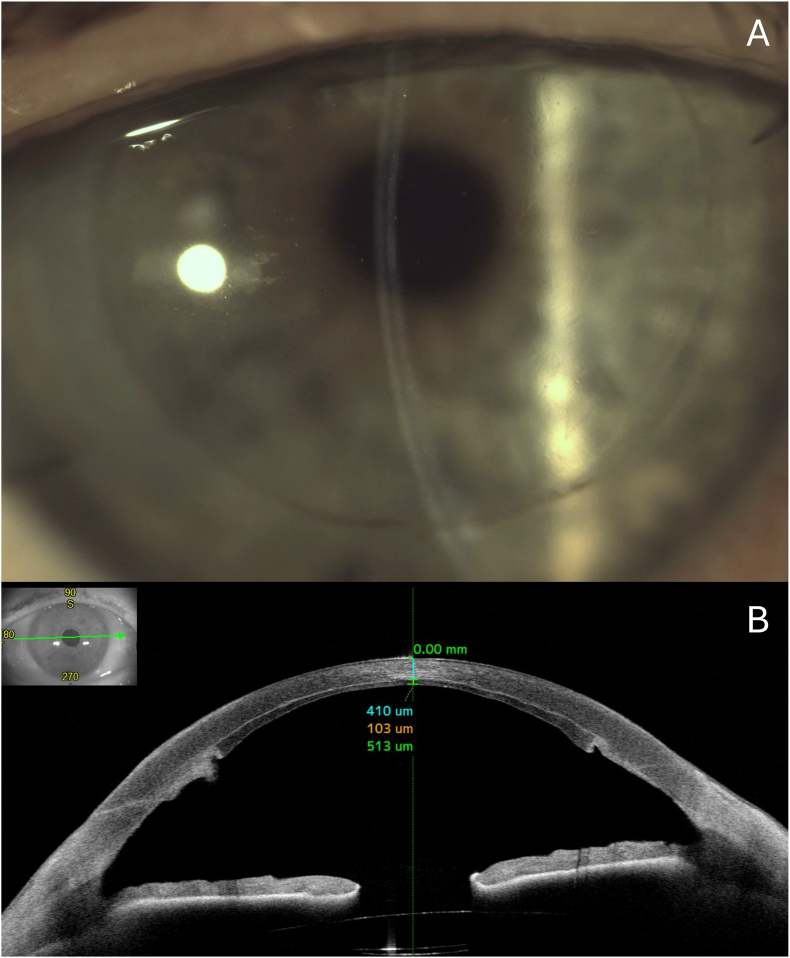
(A) Slit-lamp photograph at 3-month follow-up showing a clear cornea without residual deep stromal opacities. (B) AS-OCT confirming a well-positioned DSAEK graft with no signs of detachment, overlap or central interface haze. Central corneal thickness measured 513 μm, comprising a 103 μm-thick graft and 410 μm of overlying host stroma.

At follow-up visits, no graft detachment occurred, and the cornea progressively cleared starting one week after surgery, with no stromal haze or scarring. At final follow-up (3 months postoperatively), the patient's BCVA had improved to 0.10 logMAR. Central corneal thickness measured 513 μm, consisting of a 103 μm DSAEK graft and 410 μm of overlying host stroma. IOP remained within normal limits under current topical antiglaucoma therapy, and no graft detachment was observed (Fig. 2).

## 3 Discussion

In the present report, we describe a single-stage, femtosecond-assisted DSAEK approach for managing posterior stromal opacity in the context of suboptimal visual recovery following DMEK. This technique combines deep stromal excision and endothelial replacement in a single session, using femtosecond laser to facilitate a controlled and reproducible dissection.

Femtosecond laser technology has increasingly been integrated into corneal surgery, offering precision and customization for both donor tissue preparation and host bed dissection.^10^ Initially, the femtosecond laser has been explored primarily as a tool for improving donor preparation in endothelial keratoplasty. Femtosecond laser-assisted donor tissue preparation has been studied in the context of DSAEK to achieve thinner and more uniform grafts.^15^, ^16^, ^17^, ^18^ While early studies confirmed the ability of femtosecond systems to generate reproducible lenticules with precise depth control,^15^^,^^16^ several reports have highlighted technical limitations, including rougher stromal interfaces, greater endothelial cell loss, and interface haze, particularly with transepithelial techniques.^19^^,^^20^ The transendothelial approach offered improvements in graft regularity and thickness predictability, but concerns about ECL persisted.^17^^,^^18^ Consequently, microkeratome-assisted DSAEK remains the standard, with femtosecond donor preparation largely limited to select experimental or niche applications.^21^

Beyond DSAEK, femtosecond lasers have also been applied to DMEK, particularly to assist descemetorhexis (DR). Femtosecond-assisted DR enables consistent circular incisions, minimizes graft-host overlap, and may reduce stromal trauma.^22^ While visual outcomes appear comparable to manual DR, femto-assisted techniques have been associated with lower rebubbling rates and improved graft adherence in some studies.^11^^,^^23^

Posterior stromal scarring presents a notable barrier to successful repeat endothelial keratoplasty, often limiting visual rehabilitation and graft adherence.^7^ In such settings, replacing only the endothelium may be insufficient, and deeper tissue excision is required. While PK has historically served this role, its disadvantages have driven interest toward lamellar alternatives. Moreover, in patients with ocular hypertension or glaucoma risk factors, avoiding PK may help reduce the likelihood of postoperative intraocular pressure spikes, given the well-documented association between PK and secondary glaucoma.^7^^,^^8^

Despite its potential to selectively remove and replace diseased posterior stroma along with Descemet membrane and endothelium, DLEK never gained widespread adoption due to its technical complexity, particularly the challenge of achieving smooth, deep stromal dissections through small incisions.^1^^,^^2^ To streamline the procedure, Terry et al. proposed the use of femtosecond laser technology for anterior stromal dissection, aiming to improve reproducibility and reduce intraoperative risk.^24^ Due to technical and regulatory limitations of the time, the laser was not employed to perform the posterior vertical trephination cuts required for complete disc excision. These incisions were instead completed manually. Although this approach reduced surgical time and minimized the risk of anterior perforation, scanning electron microscopy revealed that the laser-dissected stromal interfaces were not smoother than those obtained with manual instruments. The authors concluded that optimization of deep stromal laser parameters would be necessary before broader clinical application could be considered.^24^

Alió del Barrio et al. proposed a two-stage surgical technique for managing cases of failed endothelial keratoplasty complicated by posterior stromal scarring.^25^^,^^26^ In their approach, a manual posterior lamellar dissection was performed one week prior to endothelial grafting (either DMEK or DSAEK), with the aim of creating a smoother host bed and promoting early interface remodeling before donor implantation. The rationale was based on the technical limitations of deep femtosecond stromal cuts, which often resulted in incomplete dissection due to tissue bridges and irregular surfaces, especially in edematous corneas. While effective in selected cases, this method required an additional surgical step and relied on precise manual dissection, which can be challenging and time-consuming.

Differently from the staged manual approach described by Alió del Barrio et al., the technique presented in this report leverages femtosecond laser technology to achieve a single-session, posterior stromal excision with high precision. Thanks to the intraoperative OCT-guided planning available on the FEMTO LDV Z8 platform, the dissection plane can be customized in real time based on individual corneal anatomy, enabling accurate depth control even in cases with preexisting scarring. Importantly, unlike prior methods relying on manual dissection to compensate for laser limitations at greater stromal depths, both the horizontal lamellar and vertical trephination cuts in our case were performed with the femtosecond laser, allowing en bloc removal of the posterior stromal disc. Although minor tissue bridges were observed intraoperatively, particularly in peripheral areas, these were few and easily resolved with dissection. This “en bloc excision” refers to the removal of a single, well-defined cylindrical block of posterior stromal tissue, Descemet membrane, and dysfunctional endothelium in one surgical step. In contrast, the two-stage manual approaches described by Alió del Barrio et al.^25^^,^^26^ require an initial posterior stromal dissection in one session, followed by delayed endothelial grafting in a second procedure. While their staged technique aims to enhance interface quality through pre-remodeling of the host bed, it introduces additional surgical complexity, longer recovery, and the risk of wound healing between stages. The en block strategy, by enabling simultaneous tissue removal and endothelial replacement, simplifies workflow, reduces intraoperative time, and may optimize graft-host apposition from the outset.

Compared to previous femtosecond-assisted approaches, our use of a low-energy, high-frequency laser system optimized for lamellar keratoplasty appears to significantly reduce the incidence of incomplete cuts and irregular surfaces. This likely contributed to the creation of a relatively smooth host bed, potentially favouring graft adherence and interface clarity. Moreover, to reduce the risk of donor-host mismatch or partial graft detachment, the horizontal lamellar cut was intentionally oversized by 0.50 mm relative to the DSAEK graft diameter. This configuration aims to minimize the potential for interface misalignment and prevent interface air trapping or double anterior chamber formation, complications previously reported with equal-sized donor and host cuts.^27^

Recently, Barequet et al. employed femtosecond laser technology in patients with failed DALK to enhance the precision of DR in subsequent DMEK procedures.^13^ Their technique involved a vertical cylindrical incision extending into the pre-Descemet stroma, aiming to facilitate membrane removal and improve graft-host alignment. However, stromal intervention was intentionally limited, with deep dissection explicitly avoided to preserve tissue integrity. While this approach may be suitable in cases with minimal interface alteration, it offers limited access to deeper posterior stromal changes.

In contrast, our technique incorporates both horizontal lamellar dissection and a vertical cut, enabling the removal of the diseased posterior stroma. This facilitates host bed regularization in the presence of significant scarring, potentially enhancing graft adherence and visual outcomes in more complex surgical scenarios. Additionally, the use of a posterior stromal lenticule, as in DSAEK, helps restore corneal thickness and maintain posterior curvature continuity, potentially reducing aberrations or instability that could arise when substantial stromal volume has been excised. Moreover, femtosecond-created stromal side cuts may promote wound edge healing, potentially contributing to more stable DSAEK lenticule adherence.^27^ For these reasons, we opted for DSAEK, rather than DMEK. To aid surgical decision-making, a comparative summary of key parameters across alternative approaches is provided in Table 1.

**Table 1:** Comparative summary of surgical techniques for endothelial replacement in the setting of stromal scarring or graft failure. Femto-DSAEK is presented alongside other approaches including penetrating keratoplasty (PK), standard DSAEK/DMEK, two-stage manual dissection with endothelial keratoplasty (EK), and DMEK with femtosecond-assisted descemetorhexis (Femto-DR). Key parameters include tissue removal, surgical staging, interface regularity, scarring management, graft adherence risk, and visual rehabilitation, highlighting ideal candidates and main limitations for each technique.

Parameter	Femto-DSAEK	Penetrating Keratoplasty (PK)	DSAEK/DMEK Alone	Two-Stage Manual Dissection + EK	DMEK with Femto-DR
**Tissue Removal**	Posterior stroma + DM + endothelium (laser-assisted en bloc)	Full-thickness cornea	DM + endothelium only	Manual stromal dissection followed by EK	DM + endothelium only
**Surgical Staging**	Single-step	Single-step	Single-step	Two-step	Single-step
**Interface Regularity**	High (femto-guided dissection)	None (no interface)	Variable (no stromal reshaping)	Variable (manual-dependent)	High (Femto-DR enables consistent capsulotomy-like edge)
**Scarring Management**	Direct excision of stromal opacity	Removed entirely	Not addressed	Manually excised	Not addressed
**Graft Adherence Risk**	Low (clean host bed, precise matching, undersized graft)	N/A	Increased in case of stromal haze or scarring	Variable; depends on interface quality	Low
**Risk of Immune Rejection**	Low	High (full-thickness graft)	Low	Low	Low
**Technical Demand**	Moderate to high (requires femto setup + OCT)	Moderate	Low/Moderate	High (manual precision)	High (membrane handling + femto interface)
**Visual Rehabilitation**	Fast	Slow (astigmatism, suture-related delay)	Moderate/Fast	Variable (depends on dissection quality)	Fast (if no haze)
**Use in Complex Cases**	Excellent (customizable, reproducible)	Viable but invasive	Limited by stromal opacity	Effective but complex	Limited by haze, may need conversion
**Surgical Time**	Moderate	High	Short	Cumulative high (2 procedures)	Moderate
**Main Limitation**	Technology-dependent; early experience	Graft rejection, astigmatism, glaucoma risk	Poor outcomes with stromal opacity	Two surgeries, manual variability	Unsuitable in stromal scarring
**Ideal Candidate**	Stromal haze + failed DMEK	Any severe corneal pathology	Primary endothelial disease with clear stroma	Failed EK + localized scarring	Primary endothelial disease

## 4 Conclusion

Femto-DSAEK may offer a viable alternative for managing complex cases of failed DMEK associated with posterior stromal opacity, effectively avoiding the need for PK. By combining intraoperative OCT-guided planning with a low-energy, high-frequency laser system, the technique allows for precise excision of the diseased posterior stroma and immediate endothelial replacement in a single session. This approach simplifies the surgical workflow and enables host bed regularization, potentially improving graft adherence and visual recovery, even in eyes with significant stromal scarring. Additionally, the use of a DSAEK lenticule helps restore corneal thickness and preserve posterior curvature continuity. Nevertheless, this report describes a single case, and broader clinical validation is needed. Future studies involving larger cohorts and extended follow-up are essential to assess long-term outcomes, reproducibility, and safety. Additionally, further optimization of laser parameters, particularly for deep stromal dissection, may help refine the interface quality and ensure consistent results across different corneal profiles.

## CRediT authorship contribution statement

**Luca Lucchino:** Writing – review & editing, Writing – original draft, Investigation. **Davide Romano:** Writing – review & editing, Writing – original draft. **Sabrina Vaccaro:** Investigation. **Antonio Moramarco:** Writing – review & editing. **Francesco Semeraro:** Resources. **Luigi Fontana:** Supervision. **Vito Romano:** Writing – review & editing, Supervision, Investigation, Conceptualization.

## Patient consent

Consent to publish this case report has been obtained from the patient in writing.

## Declaration of generative AI and AI-assisted technologies in the writing process

During the preparation of this work, the author(s) used ChatGPT (OpenAI, San Francisco, CA, USA) to assist with language refinement and improve clarity and grammar. After using this tool, the author(s) reviewed and edited the content as needed and take full responsibility for the content of the publication.

## Funding

This research did not receive any specific grant from funding agencies in the public, commercial, or not-for-profit sectors.

## Declaration of competing interest

The authors declare that they have no known competing financial interests or personal relationships that could have appeared to influence the work reported in this paper.
