# Supplementary material for: Femto-DSAEK for deep posterior stromal opacity: a lamellar alternative to PK in failed DMEK
Source: Am J Ophthalmol Case Rep. 2026 Mar 2;42:102552. doi: 10.1016/j.ajoc.2026.102552 (PMC12996227; doi:10.1016/j.ajoc.2026.102552)
Supplement: Multimedia component 1 [file mmc1.docx]

**Supplementary Video 1.** Intraoperative video showing DSAEK graft insertion and positioning after femtosecond-assisted posterior stromal cut.
